# Relationship between the Quality of Colostrum and the Formation of Microflora in the Digestive Tract of Calves

**DOI:** 10.3390/ani10081293

**Published:** 2020-07-29

**Authors:** Kamila Puppel, Marcin Gołębiewski, Katarzyna Konopka, Małgorzata Kunowska-Slósarz, Jan Slósarz, Grzegorz Grodkowski, Tomasz Przysucha, Marek Balcerak, Beata Madras-Majewska, Tomasz Sakowski

**Affiliations:** 1 Institute of Animal Science, Warsaw University of Life Sciences, Ciszewskiego 8, 02-786 Warsaw, Poland; kamila_puppel@sggw.edu.pl (K.P.); marcin_golebiewski@sggw.edu.pl (M.G.); konopka.katarzyna96@gmail.com (K.K.); malgorzata_kunowska_slosarz@sggw.edu.pl (M.K.-S.); jan_slosarz@sggw.edu.pl (J.S.); grzegorz_grodkowski@sggw.edu.pl (G.G.); tomasz_przysucha@sggw.edu.pl (T.P.); marek_balcerak@sggw.edu.pl (M.B.); beata_madras_majewska@sggw.edu.pl (B.M.-M.); 2 Department of Animal Science, Institute of Genetics and Animal Breeding, Polish Academy of Sciences, Jastrzębiec, Postępu 36A, 05-552 Magdalenka, Poland

**Keywords:** colostrum, calves, immunoglobulin, intestinal microflora

## Abstract

**Simple Summary:**

Colostrum is a source of antibodies and immunostimulatory components; however, it is also a factor that guarantees the rapid multiplication of microorganisms in the digestive system, which significantly affects the proper functioning of the calf’s body. The aim of the study was to determine the relationship between the quality of colostrum and the formation of intestinal bacterial microflora in calves from birth to day 7. The study showed a significant influence of colostrum quality class on the formation of the intestinal microbiome and the daily weight gains of calves. The quality of the colostrum influences the intestinal microbiome. The higher the concentration of bioactive components, the more probiotic bacterial strains can develop.

**Abstract:**

The aim of the study was to determine the relationship between the quality of colostrum and the formation of intestinal bacterial microflora in calves from birth to day 7. Seventy-five multiparous Polish Holstein–Friesian cows were selected. Colostrum samples were collected individually up to two hours after calving. The analysis was carried out on 75 calves; which were divided into three groups based on the colostrum quality class of the first milking. Faecal samples were collected rectally from each calf on its seventh day of life. Calves were weighed twice; on days 0 and 7 of life. It has been shown that with a higher concentration of colostrum protein fraction, primarily immunoglobulins, the colonisation of anaerobic bacteria occurs faster. Colostrum with a density >1.070 g/cm^3^ promoted the significant development of *Lactobacilli* and *Bifidobacterium* spp. which at the same time contributed to the reduction of unfavourable microflora, such as *Coliforms* or *Enterococci*. Regardless of the initial body weight, daily weight gains were highest for calves fed with colostrum with a density >1.070 g/cm^3^. The study showed a significant influence of colostrum quality class on the formation of the intestinal microflora and the daily weight gains of calves.

## 1. Introduction

Bovine colostrum has three classes of immunoglobulins (Ig)—A, M, and G [1,2]. In the first hour after calving, the IgG concentration may reach ~80 g/L, and thus constitutes over 85% of Ig. In contrast, IgM and IgA may constitute just ~7%, with concentration values of 8 g/L and 4 g/L, respectively [3]. At an average intestinal absorption of immunoglobulins (20–30%), the calf should consume 100–200 g of IgG within six hours after calving. This guarantees an adequate passive transfer [4].

The concentration of lactoferrin (LF) in colostrum is relatively high. The average concentration for Holstein cows ranges from 1.2 and 3.0 g/L [5], while in milk it is only 0.12–0.13 g/L [6]. It is the most important whey protein, which forms complexes with iron. In juveniles, there is no intestinal barrier in the initial period, therefore LF has the possibility of remaining for a longer period and consequently penetrating into the bloodstream and controls the proper composition of the gut microflora. LF affects cell proliferation, including that of epithelial cells of the small intestine and colon, and it is also a factor determining the multiplication of *Lactobacilli* and *Bifidobacteria* [7]. It has a system to combat pathogenic microorganisms: it destroys enzymes and bacterial structures, such as fimbria, which allow colonisation and adhesion to the walls of the intestine, thus reducing the risk of bacteraemia or endotoxemia [7].

α-lactalbumin (α-LA) and β-lactoglobulin (β-LG) are globular proteins that account for up to 80% of the total weight of whey proteins [8]. The concentration of β-LG in colostrum for the Holstein breed is 6 g/L [9], while that of α-LA may range from 5 g/L [10] to even 8 g/L [11]. As reported by Caffin et al. [12], the concentrations of these whey proteins depend on each other—there is a positive correlation. They affect milk secretion processes [12] and are indirectly responsible for the concentration of selected ingredients. α-LA can bind metal ions, e.g., calcium, cobalt, magnesium, or zinc [13], while β-LG may be responsible for the transport of hydrophobic molecules, e.g., vitamins, fatty acid metabolism. β-LG, by reducing the colonisation of *Staphylococcus aureus* and *Streptococcus uberis*, minimises the risk of mastitis in the herd. Additionally, the antimicrobial activity against *Staphylococcus aureus* and *Streptococcus uberis* is concentration dependent and elicited by the intact protein [14]. Both β-LG and α-LA have anti-cancer, antioxidant, antiviral, and antibacterial activity [8,9].

Colostrum is a source of antibodies and immunostimulatory components; however, it is also a factor that guarantees the rapid multiplication of microorganisms in the digestive system, which also significantly affects the proper functioning of the calf’s body [15]. The beneficial microflora produced by fermentation in the rumen approximately 70% of a calf’s daily energy demand, due to the recalcitrant nature of structural carbohydrates, such cellulose, xylans, mannans, pectins, inulin, and beta glucans [16]. In addition, it influences feed intake, it shapes the future productivity of the animal [17]. It is also necessary in creating a physical barrier in the digestive system—thanks to microflora, it is possible for the animal to secrete intestinal mucus and maintain the proliferation of cells that contribute to the reconstruction of the barrier after possible injuries [18]. In addition, it is essential for the development of lymphatic structures associated with the intestinal mucosa, which determines the body’s immune response. According to Sommer and Backhed [19], this task is assigned to Peyer’s patches. More than 60% of the cells responsible for immunisation of the body are connected with the intestinal submucosa [20]. The proper development of the mucous membrane and the supply of specific antigens during prenatal life affect the production and secretion of IgA [21].

The number of the microbiota in the digestive system depend on many factors. Malmuthuge et al. [22] divided these factors into three components: (i) Those that are host-dependent (e.g., food retention in the intestine and defence mechanisms of organisms), (ii) microorganisms (e.g., their adhesion and mechanism of obtaining ingredients or behaviour in different oxygen gradients), and (iii) the external environment (e.g., the maternal microbiome, birth hygiene, diet, and treatment). It has been noted that in younger animals, both the variety and density of colonisation is higher than older animals [23]. A similar relationship has been found in calves that have experienced disease, e.g., diarrhoea or pneumonia [24]. Dominguez-Bello et al. [25], by testing samples of calf faeces 24 h after calving, were able to determine how the digestive system was colonised. Research shows that the microbiological composition of meconium is very similar to that of the maternal birth canal. In the initial stages of the gastrointestinal tract’s development, the colonies that are formed vary greatly and include both aerobic and anaerobic bacteria. The initiators of colonisation are primarily *Staphylococcus*, *Streptococcus*, *Enterococcus*, and *Enterobacteria*. Using oxygen, they create an anaerobic environment in further stretches of the intestine [22]. With the administration of colostrum, lactic acid bacteria, i.e., *Lactobacillus*, *Bifidobacterium spp.*, *Enterococcus,* and *Pediococcus* spp., spread in a very short time; they ferment sugars and thus contribute to the production of acids, e.g., lactic or propionic [26]. In addition, these bacteria have a therapeutic function, as some of them inhibit pathogens, e.g., *E. coli*, which also colonise very quickly in the calves’ digestive system.

The *Coliforms* group includes species of the genus *Klebsiella*, *Enterobater*, rods of the *Enterobacteriaceae* family, and the most well-known, *E. coli*. These are called environmental pathogens [27], but they have ability to multiply in the digestive systems of warm-blooded animals [28]. According to Moxley and Francis [29], they accumulate most in the mucosa of the large intestine and ileum. They are capable of rapid invasion in the body. According to Smith’s studies on calves, *E. coli* can be identified as early as within eight hours after birth, while *Lactobacillus* strains appear on the first day of a calf’s life [30]. These bacteria can produce cytotoxins and are attacked Peyer’s patches, disturbing the action of the faecal cells; this is followed by an increase in the population of the microorganisms concerned. According to research by Tannock [31], the decrease in the occurrence of lactic acid bacteria strains is caused by the growth of *Coliforms*. In people with Crohn’s disease, the presence of *E. coli* in the intestinal mucosa exceeds 50% of the whole microbiome, while in healthy people, it is only 1% [32]. According to Hamound et al. [33], *E. coli* is one of the main factors in calf mortality. Almost half of the examined animals that died within one month after birth were positive for the presence of this bacterium in the body. *E. coli* causes colibacillosis and sepsis. The most susceptible seem to be 1- to 3-day-old calves, as well as 3- to 8-week-old calves. Clinical symptoms of *E. coli* colonisation include diarrhoea, increased body temperature, general weakness, and dehydration [34].

*Bifidobacterium* spp. and lactic acid bacteria given to calves in the first week of life have a positive effect on weight gain, result in better feed utilisation, and minimise the risk of diarrhoea [35]. It has been observed that a higher density of these bacteria in the intestine is correlated with a lower incidence of intestinal diseases, which is associated with the maintenance of adequate intestinal homeostasis [36]. *Bifidobacterium* spp. have a stabilising effect on the formation of an immune response. This effect is very important in the processes of T cell differentiation and proliferation [37]. Godden et al. [38] reported that these bacteria, together with other microorganisms, improve IgG absorption and form an intestinal barrier.

*Lactobacillus* bacteria are considered to be particularly beneficial because they have the ability to break down proteins, carbohydrates and fats, and they also contribute to the better absorption of nutrients. *Lactobacillus* bacteria strains produce organic acids, hydrogen peroxides, enzymes, and bacteriocins [39]. In the calf’s body, *Lactobacillus* colonise very quickly—within 24 h after birth [30], and like *Bifidobacteria* spp., *Lactobacilli* are the dominant bacteria in the digestive system during the calf’s first week of life. Their colonisation may be affected by pH (close to neutral), and the presence of oxygen [40].

The aim of this study was to determine the relationship between the quality of the colostrum and the formation of intestinal bacterial microflora in calves in their first week of life. The following work analyses the chemical composition of the colostrum (whey protein fraction) and bacterial count (total anaerobic bacteria, *Lactobacilli*, *Bifidobacterium* spp., *Coliforms*, and *Enterococci*). The study also analyses the daily weigh gain of calves in the first week of life. This information made it possible to demonstrate how high- and low-quality colostrum shape the intestinal microflora and the relationship between the quality of colostrum and daily weight gain in calves.

## 2. Materials and Methods

All cows were handled in accordance with the regulations of the Polish Council on Animal Care, and the Second Ethics Committee for Animal Experimentation in Warsaw reviewed and approved all procedures (Approval number: WAWA2/086/2018). During the study, the cows were under veterinary care and did not show any disorders or diseases. The research was conducted on an experimental organic dairy farm, in which a herd of approximately 250 cows was kept in a freestall housing system, with the average production exceeding 6000 kg of milk per lactation. Dry cows were fed according to the guidelines of the Nutrient Requirements Committee (Table 1).

Seventy-five multiparous (in second and third lactation) Polish Holstein–Friesian cows were selected for the experiment. An additional criterion was the production of at least 2 L of colostrum in the first milking. The colostrum yield was medium (3–6 kg) for all cows, and did not exceed 6 kg/milking. Colostrum samples (250 mL) were collected individually up to two hours after calving, placed in sterile bottles and transported to the Warsaw University of Life Sciences for analysis- all analyses were carried out in fresh colostrum.

The analysis was carried out on 75 calves (of both sexes: 51 ♀ and 24 ♂), which were divided into three groups based on the colostrum quality class of the first milking: 1st class colostrum density >1.070 g/cm^3^ (very good colostrum); 2nd class colostrum density 1.057–1.070 g/cm^3^ (good colostrum); and 3rd class colostrum density 1.044–1.056 g/cm^3^ (sufficient colostrum). The calves were removed from their dams and housed in individual pens. They were fed with colostrum/milk for five days. Colostrum was served individually four times a day (1–1.5 L/feeding). After the initial feeding of colostrum, for the next three days calves receive transition milk from their dam, three times per day (1.5–2.5 L/feeding). The calves had continuous access to water (from the first day of life), calf starter, and hay (from the fifth day of life). No calves were given antibiotic treatment prior to faecal sampling.

### 2.1. Microbiological Assay

Faecal samples (with no lubricant used, in aseptic condition) were collected rectally from each calf on day 7 of life and immediately transferred to a test tube filled with Wilkins–Chalgren broth (Sigma-Aldrich, St. Louis, MO, USA). Next, samples were serially diluted in the same medium. Appropriate dilutions (1 mL) were transferred to sterile 90 mm Petri dishes. MRS agar (Sigma-Aldrich, St. Louis, MO, USA) was used to study *Lactobacilli* (*L. brevis*, *L. fermenti*) and *Bifidobacterium* spp. Reinforced Clostridial Medium (Sigma-Aldrich, St. Louis, MO, USA) was used to study *Clostridium* spp. and other anaerobic microorganisms. MacConkey agar (Sigma-Aldrich, St. Louis, MO, USA) was used to study *Coliform* bacteria and *Enterococci*. *Bifidobacterium* spp. were incubated in anaerobic conditions at 37 °C for 72 h. The *Lactobacilli* were incubated aerobically at 37 °C for 48 h. Petri dishes with MacConkey agar were incubated aerobically at 37 °C for 24 h to detect *Coliform* bacteria or 48 h to detect *Enterococci*.

Moreover, calves were weighed twice: On days 0 (before the first colostrum feeding) and 7 of life.

### 2.2. Chemical Analyses of Colostrum

The microbiological quality of colostra was determined by *BactoScan* (Bentley Instruments, MN, USA). It should be noted that the colostrum samples met the microbiological quality requirements for bacterial contamination (<100,000 CFU/mL).

The basic chemical composition of the colostrum, i.e., fat, protein, casein, density, and lactose content, was determined by automated infrared analysis using a MilkoScan FT–120 analyser (Foss Electric, Denmark).

Concentrations of whey proteins were determined using an Agilent 1100 Series RP-HPLC (Agilent Technologies, Waldbronn, Germany) according to the methodology described by Puppel et al. [41]. Separations were performed at ambient temperature using a solvent gradient on a Jupiter Column C18 300A (Phenomenex, Torrance, CA, USA). The chromatographic conditions were as follows. Solvent A was acetonitrile (Merck, Darmstadt, Germany), water (Sigma–Aldrich, St. Louis, MO, USA), and trifluoroacetic acid (Sigma-Aldrich, St. Louis, MO, USA) in a ratio of 50:950:1 (v/v/v). Solvent B was acetonitrile, water, and trifluoroacetic acid in a ratio of 950:50:1 (v/v/v). The flow rate was 1.2 mL/min, and the detection wavelength was 220 nm. The injection volume of the final solution was 25 μL. All samples were analysed in duplicate. The identification of peaks as those of LF and lysozyme was confirmed by a comparison with the standards: LF and LZ (Sigma-Aldrich, St. Louis, MO, USA).

Concentrations of immunoglobulins were determined using an Agilent 1100 Series RP-HPLC (Agilent Technologies, Waldbronn, Germany) according to the methodology described by Puppel et al. [41]. Separations were performed using a solvent gradient on a Jupiter Column C18 300A (Phenomenex, Torrance, CA, USA). The chromatographic conditions were as follows. Solvent A was acetonitrile (Merck, Darmstadt, Germany), water (Sigma–Aldrich, St. Louis, MO, USA), and trifluoroacetic acid (Sigma–Aldrich, St. Louis, MO, USA) in a ratio of 10:990:1 (v/v/v). Solvent B was acetonitrile, water, and trifluoroacetic acid in a ratio of 990:10:1 (v/v/v). The column was first equilibrated at 25% mobile phase A for 2 min at a 2 mL/min flow rate. The elution was performed as a gradient of mobile phase A, from 25% to 60% over 5 min at 2 mL/min. The detection wavelength was 280 nm. The injection volume of the final solution was 25 μL. All samples were analysed in duplicate. The identification of peaks as immunoglobulins was confirmed by a comparison with the standards of bovine IgG (Sigma-Aldrich, St. Louis, MO, USA).

### 2.3. Statistical Analyses

The experimental data were statistically analysed using SPSS 23 [42]. Data were presented as least squares means with standard error of the mean.

The statistical model was:

Y_ijk_ = µ + A_i_ + e_ij_

where: y is the dependent variable, µ is the overall mean, A_i_ is the fixed effect of the colostrum quality (i = 1–3), and e_ijk_ is the residual error.

After preliminary analyses, the calves were divided into three groups based on the colostrum quality class of their first milking:

1st class colostrum density 1.070 g/cm^3^: 23 calves
2nd class colostrum density 1.057–1.070 g/cm^3^: 25 calves
3rd class colostrum density 1.044–1.056 g/cm^3^: 27 calves

## 3. Results and Discussion

The quality classification of the colostrum is directly related to its density. An insufficient concentration of bioactive components is responsible for lowering the colostrum’s immunostimulatory properties. A density > 1.050 g/cm^3^ is considered to be good [43]. In this study, the term “good” was assigned to the 2nd class, with a density of 1.057–1.070 g/cm^3^, at over 22% protein and 7% fat (Table 2). “Very good” (1st class) colostrum had a density value > 1.070 g/cm^3^, and the density of “sufficient” (3rd class) colostrum fell within the range of 1.044–1.056 g/cm^3^. Wroński and Sosnowska [44], who also conducted research on the Holstein breed, reported slightly lower values. For comparison, the density of the “very good” class had an average value of 1.054 g/cm^3^, with approximately 18% protein and 5.5% fat.

In accordance with the findings of Pecka et al. [45], we found that the colostrum density was positively correlated primarily with the components of the protein fraction, which contains the most important immune components affecting the health of the animal. On the basis of the obtained results, a significant relationship between the colostrum class and the level of bioactive whey proteins was demonstrated (Table 3). The concentrations of LF, α-LA, and β-LG in the 1st class colostrum were higher than the 3rd class colostrum by almost 100%, and the concentrations of immunoglobulins were almost 200% higher.

**Figure 1:**
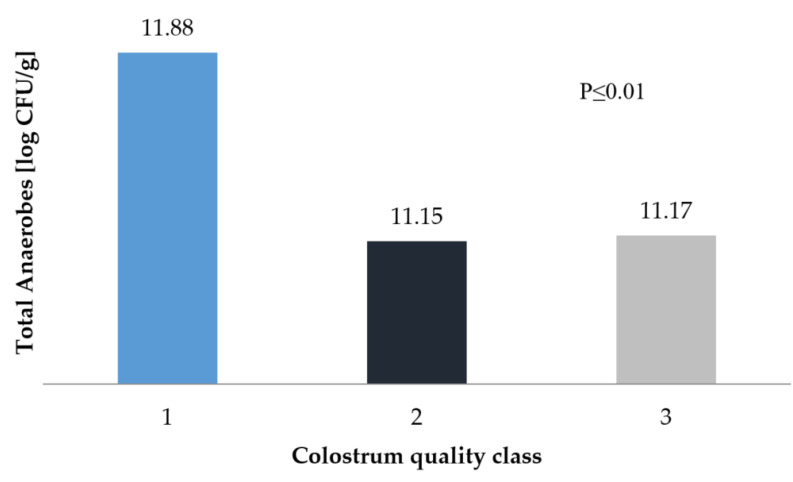
Total anaerobes count in faecal samples depending on the class of colostrum.

In our study, the total anaerobic bacteria count for the highest class of colostrum was 11.88 log CFU/g, while the bacteria count in calves fed with colostrum of lower classes was 11.15 log CFU/g (Figure 1). According to Jami et al. [23], the digestive system of a 3-day-old calf is inhabited by similar bacterial strains as that of an adult. The study shows how the composition of the microflora of the digestive system of cattle in different technological groups changes. In adults, approximately 51% of bacteria are *Bacterioidetes* and approximately 42% are *Firmicutes*, while in newborns, *Poteobacteria* prevails. Our own research shows that the quality of colostrum has a very significant influence on the colonisation of bacteria in the digestive system. Hyrslova et al. [46] also claim that strain growth depends on high levels of bioactive components, such as LF, lysozyme, or lactoperoxidase, which help to maintain the balance of the intestinal microflora. A high quality of colostrum is associated with the rapid development of anaerobic or optionally anaerobic bacterial strains, which are primarily responsible for the composition of complex in the intestine into simple substances. As reported by Fonty et al. [47], on the second day of life, the number of anaerobes reaches 109 CFU/mL of rumen fluid.

The total anaerobes reported by Minato et al. [48] ranged from 8.3 log CFU/g on the first day of a calf’s life to 9.3 log CFU/g on day 7. However, the samples were taken directly from the rumen and the calves were fed with colostrum until they were three days old; moreover, the colostrum was not subjected to detailed chemical tests. Based on the same methodology, similar results were obtained by Anderson et al. [49]. After one week, they obtained 10^−9^ CFU/g anaerobic bacteria and 10^−7^ CFU/g anaerobic bacteria. The results most similar to our own were presented in the work of Teraguchi et al. [50]. However, in their study, bacterial colonisation of the digestive system was studied in mice given cows’ milk, which differs significantly from colostrum in its chemical composition. The anaerobic bacteria colony on the first day was 9.8 log CFU/g and on the seventh day, 11.0 log CFU/g.

The environmental niche in the rumen changes very quickly during the first three days of a calf’s life. The most noticeable changes are the reduction of aerobic bacteria and the growth of anaerobic bacteria. The dynamics of the development of primary populations in this postnatal time is very important for the formation of the rumen microflora of adult animals [23]. Calves fed with poor-quality colostrum may have a problem with poor absorption of nutrients, which may result in poorer health and reduced productivity later in life. Colostrum of poor quality may cause slower colonisation of anaerobic strains. In a comparison of faecal samples from a newborn vs. a 3-day-old calf, an increase in *Ruminococcus* (*R. flavefaciens* and *R. albus*) was found [51].

**Figure 2:**
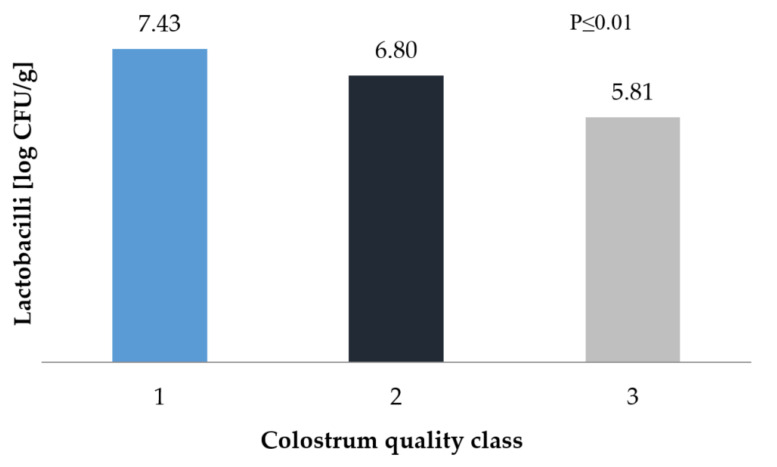
*Lactobacilli* count in faecal samples depending on the class of colostrum.

A high quality of colostrum has been shown to be positively correlated with the growth of the probiotic microbiome. Regardless of the colostrum class, the development of *Lactobacilli* strains was quite good. Rapid colonisation is related to the fact that these bacteria are supplied with colostrum/milk, so during the first weeks of life, their levels in the digestive system tend to increase. According to Oikonomou et al. [24], the highest count of colonies, of both *Lactobacillus* and *Bifidobacterium spp.*, occurs in the fourth week of life (14.74% of bacteria), which then decreases (in the seventh week of life, it is only 2.15%). In our work, it can be seen that the higher the colostrum class, results the higher count of the strain. The *Lactobacilli* were within the limit of approximately 5 to 7 log CFU/g (Figure 2). Similar values in bacteria count have been reported by Minato et al. [48] and Anderson et al. [49]. In contrast, Higginbotham and Bath [52] recorded a colony count exceeding 8.5 log CFU/g already on the first day of the calf’s life. Ellinger et al. [53] fed cows fermented colostrum (dry matter content: 15.5%, fat content: 4.92%, protein content: 4.90%, *Lactobacilli* content: 5.56 log CFU/g) and averaged the results from three faecal samples (obtained after 7, 14, and 21 days); with these parameters, 6.93 log CFU/g was achieved, which equates to a diet based on high-quality colostrum in own work.

**Figure 3:**
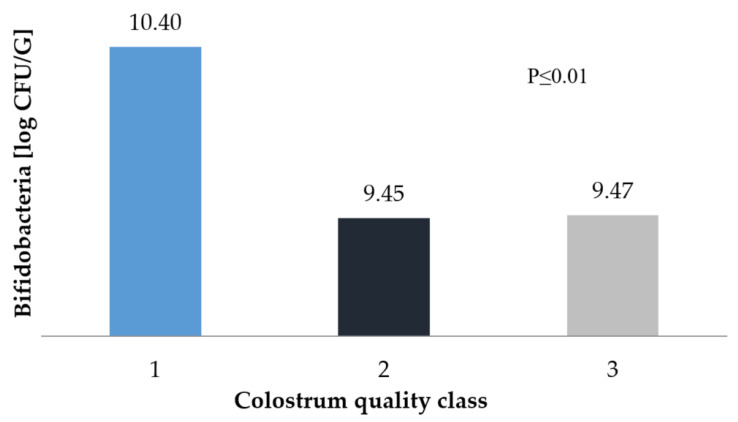
*Bifidobacteria* count in faecal samples depending on the class of colostrum.

Differences were found in the case of *Bifidobacterium* spp. colonisation in the calves’ digestive system. Colostrum with a density <1.070 g/cm^3^ is associated with weaker development of the studied strains. The difference in results between the classes of colostra was more than 1 log CFU/g¸ with the highest value of approximately 10.4 log CFU/g for 1st class colostrum (Figure 3).

Colostrum, which affects the formation of the number of strains above 9.0 log CFU/g from the faecal sample, can be compared to the action of regular milk or colostrum subjected to various technological processes, which have less influence on the development of *Bifidobacterium spp.* Vlková et al. [54], using a milk-based diet for calves, showed an increase in bacteria count from 7.6 log CFU/g on day 4 to 9.2 log CFU/g on day 11. In contrast, Hyrslova et al. [55] studied the effect of pasteurised and cooled bovine colostrum on the development resulted in *Bifidobacterium* spp. was at the level 7 of log CFU/g. These examples show how important it is to guarantee the quality of colostrum in order to influence the development of microflora, which affects many different processes, such as increasing IgG concentration. Fonty et al. [47] and Malmuthuge et al. [22] showed a negative correlation between the expansion of *Bifidobacterium* and *E. coli*. This relationship was confirmed in this study.

The presence of *Coliforms* in the herd can prove to be very dangerous, as it contributes to an increase in the mortality of young animals, so it is important that their count is as low as possible. This effect can be achieved by feeding calves colostrum with a high concentration of immunostimulatory components. According to Johnson et al. [56], high temperature inhibits *E. coli* growth. Heat treatment of colostrum leads to a two-fold reduction in the pathogenic microflora count (from approximately 3.7 to 1.2 log CFU/g). Gilliland et al. [57] and Malmuthuge et al. [22], as with *Bifidobacterium*, showed a negative correlation between *Lactobacilli* and *Coliforms*. The addition of *L. acidophilus* NCFM to the milk limited the multiplication of pathogenic bacteria from 9.67 on the first day, the count of *Coliforms* dropped to 8.67 log CFU/g on 21st day. In contrast, in the control sample, without the addition of *Lactobacilli*, the count of bacteria decreased from 9.52 to 9.21 log CFU/g.

**Figure 4:**
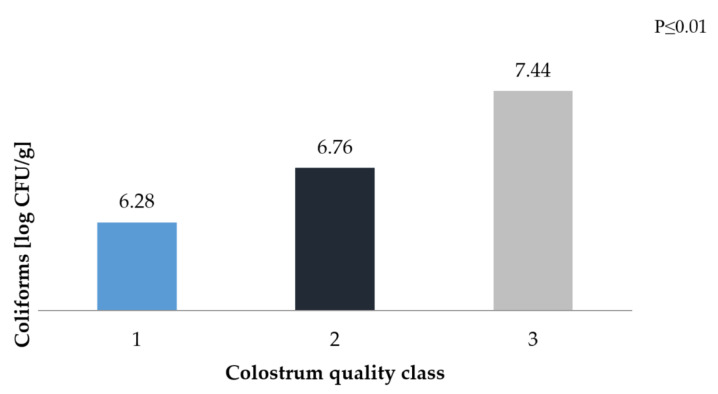
*Coliforms* count in faecal samples depending on the class of colostrum.

In our own work, colostrum of a good and sufficient class shaped the level of *Coliforms* in the range from 6.5 to almost 7.5 log CFU/g (Figure 4).

**Figure 5:**
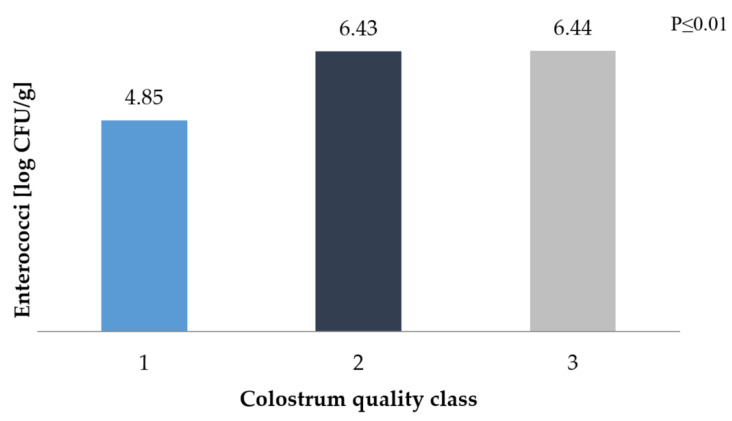
*Enterococci* count in faecal samples depending on the class of colostrum.

The poor quality of colostrum also increased the *Enterococci* count. These bacteria are commonly found in faeces and can constitute up to 1% of the mass of microorganisms (in the digestive tract, there are approximately 105–107 cells per 1 g). Their metabolism is based on the fermentation of sugars, which may have a beneficial effect on the development of the digestive system; however, a larger *Enterococci* population has a destructive effect on the body [26]. In the good quality colostrum group of calves, the level of these bacteria in the faeces was above 4 log CFU/g, while in the case of colostrum with a density < 1.070 g/cm^3^, the *Enterococci* count remained at 6 log CFU/g (Figure 5). Gregory et al. [58] reported a negative correlation between *Enterococci* and *E. coli* in the rumen. In our own work, it can also be stated that *Coliform* has an advantage in count over *Enterococci*.

The influence of colostrum quality is of great importance not only for the composition of the intestinal microflora but also for the development of the whole organism. Regardless of the initial body weight of the newborn, the daily weight gains’ were highest for calves fed with colostrum with a density >1.070 g/cm^3^, which increases the probiotic count and decreases the count of pathogenic bacteria. The difference between the fastest and slowest growing animals was 0.278 kg per day (Table 4), which suggests that calves develop twice as fast between these groups. Such differentiated weight gain can be caused by less caloric food, which is related to the content of all components, and also by the relationship between bacteria and the absorption of immunoglobulins, which guarantees the health of animal. For instance, Godden et al. [38] showed a negative effect of Coliforms on the decreasing number of immune bodies. Suo et al. [55] also mentions that bacteria, i.e., Lactobacilli, increase IgG concentration.

## 4. Conclusions

The colonisation of bacteria that are important in adult animals already begins in the first days of postnatal life, when the calf is fed only with colostrum and milk. The colostrum quality class affects both the composition of the intestinal microflora and the development of the daily weight gain. Regardless of the initial body weight, daily weight gains were highest for calves fed with colostrum whose density was >1.070 g/cm^3^.

*Lactobacilli* count depends on the amount of immunoglobulins in colostrum. There are negative correlations between *Bifidobacterium* spp. and *E. coli*, between *Lactobacilli* and *Coliforms*, and between *Coliforms* and *Enterococci*.

The higher the concentration of bioactive components, the more probiotic bacterial strains can develop. The study showed a significant influence of colostrum quality class on the formation of the intestinal microbiome and daily weight gain of calves.

## Figures and Tables

**Table 1:** Daily requirements of the cows.

Specification	Dry Cow Groups	
	I	III
Assumptions		
Cow weight (kg)	640	675
Pregnancy (days)	220	270
Maintenance requirements		
NEL (Mcal/day)	9.8	11.6
Metabolic protein (g/day)	463	653
Ca (g/day)	11	16.5
P (g/day)	12	16.3
K (g/day)	54	55
Foetus requirements		
NEL (Mcal/day)	2.9	3
Metabolic protein (g/day)	239	245
Ca (g/day)	4	5
P (g/day)	3	4
K (g/day)	1	2
Total requirements		
NEL (Mcal/day)	12.8	14.4
Metabolic protein (g/day)	700	901
Ca (g/day)	15	21.5
P (g/day)	15	20.3
K (g/day)	55	57

NEL = Net milk-producing energy.

**Table 2:** The division of cows’ colostrum into classes, taking into account the basic parameters: Casein, protein, and fat.

Colostrum Class	Density [g/cm^3^]	Casein [%]	Protein [%]	Fat [%]
**1st**	>1.070	9.60 ^aB^	28.42 ^AB^	7.29 ^AB^
**2nd**	1.057–1.070	8.53 ^ac^	22.39 ^AC^	6.64 ^Ac^
**3rd**	1.044–1.056	7.39 ^Bc^	18.22 ^BC^	5.12 ^Bc^

aa, AA: Means in the same column marked with the same letters differ significantly at: Small letters-*P* ≤ 0.05; capitals-*P* ≤ 0.01. Data are presented as LSM.

**Table 3:** The level of LF, α-LA, β-LG and IgG depending on the class of cows’ colostrum.

Colostrum Class	LF [g/L]	α-LA [g/L]	β-LG [g/L]	IgG [g/L]
**1st**	4.40 ^AB^	7.52 ^AB^	8.04 ^AB^	92.00 ^AB^
**2nd**	3.03 ^Ac^	4.55 ^AC^	6.16 ^AC^	74.60 ^AC^
**3rd**	2.53 ^Bc^	3.47 ^BC^	4.8 ^BC^	53.50 ^BC^

aa, AA: Means in the same column marked with the same letters differ significantly at: Small letters-*P* ≤ 0.05; capitals-*P* ≤ 0.01. Data were presented as LSM.

**Table 4:** Changes in calf weight depending on the cows’ colostrum class.

Colostrum Class	Weight [kg]		Daily Weight Gain [kg/day]
	Day 0	Day 7	
**1st**	40.17	43.45	0.468 ^AB^
**2nd**	41.67	43.23	0.222 ^A^
**3rd**	42.17	43.50	0.190 ^B^

AA: Means in the same column marked with the same letters differ significantly at capitals—*P* ≤ 0.01. Data were presented as LSM.
